# Slow sodium-channel inactivation underlies spike threshold variability

**DOI:** 10.1186/1471-2202-14-S1-P322

**Published:** 2013-07-08

**Authors:** Paul M Harrison, Mark J Wall, Magnus JE Richardson

**Affiliations:** 1 MOAC DTC, University of Warwick, Coventry, CV4 7AL, UK; 2 School of Life Sciences, University of Warwick, Coventry, CV4 7AL, UK; 3 Warwick Systems Biology Centre, University of Warwick, Coventry, CV4 7AL, UK

## 

Action potentials are triggered once a neuron has received sufficient synaptic input to be depolarised above threshold; however, this threshold is dynamic and strongly correlated to the recent voltage history and time since the last spike. Previous experimental measurements from cortical layer-5 pyramidal neurons have shown that after an action potential the threshold can jump up to 10 mV and subsequently decays exponentially back to its baseline value over a period of tens of milliseconds [1,2]. Our recent experimental data indicate that this behaviour is a common feature of neocortical excitatory cells in different cortical layers.

One mechanism that modulates the spike threshold is the degree and duration of sodium-channel inactivation. In the standard Hodgkin-Huxley formulation of the action potential only fast inactivation is present, with a return to the baseline state within a few milliseconds post-spike. However, a slow component of sodium inactivation is also known to be present in neocortical cells [3] with a time constant - of the order of tens of milliseconds - that is similar to the experimentally measured threshold decay.

To model the influence this mechanism has on spike threshold we added a slow sodium inactivation variable to the sodium current of an existing conductance-based model [4]. We found that the augmented model now exhibited a decaying threshold that was not present in the original model, with similar properties to that observed experimentally. An analysis of the spike-triggered slow-inactivation variable dynamics demonstrated that it could be accurately described by two parameters: the post-spike level of inactivation and the time constant of its decay back to an activated state. These gating variable parameters could in turn be directly related to the corresponding magnitude of the post-spike increase of the threshold and the time constant of its decay to baseline.

We next added this mechanism to the Exponential Integrate-and-Fire model [5] to better quantitatively understand the role of slow inactivation in modifying spike behaviour under conditions of stochastic synaptic drive at both the neuronal and network levels. Though in general this is a two variable system comprising of voltage and the post-spike threshold dynamics, we show that if the membrane and threshold-relaxation time constants are the same the system can be reduced to an effective one-dimensional model amenable to standard Fokker-Planck methods [6]. Finally, a perturbative approach is introduced that allows for the calculation of experimentally relevant quantities like the first-passage-time density and spike-train spectra for the general case where the two time constants differ.
